# Dynamic Regulation of Hypothalamic *DMXL2*, *KISS1*, and *RFRP* Expression During Postnatal Development in Non-Human Primates

**DOI:** 10.1007/s12035-016-0329-x

**Published:** 2016-12-12

**Authors:** Fazal Wahab, Charis Drummer, Stefan Schlatt, Rüdiger Behr

**Affiliations:** 1 Platform Degenerative Diseases, German Primate Center, Kellnerweg 4, 37077 Göttingen, Germany; 2 Institute of Reproduction and Regenerative Biology, Centre of Reproductive Medicine and Andrology, Albert-Schweitzer-Campus 1, Building D11, 48149 Münster, Germany

**Keywords:** Puberty, Marmoset, Non-human primate, DMXL2, Kisspeptin, Kiss1r, GnIH, GnRH, Hypothalamus

## Abstract

The neurobiological mechanism of puberty onset in primates is currently only partly understood. A recent study reported an important role of *Dmx-like 2* (*DMXL2)*, a gene encoding rabconnectin-3α vesicular protein, in human subjects with mental retardation and neuroendocrine impairment of reproduction. To further characterize the potential role of *DMXL2* in the regulation of reproduction, we analyzed the expression of *DMXL2* in hypothalami of newborn, infantile, juvenile, pubertal, and postpubertal female and male common marmoset monkeys. Additionally, as the relative hypothalamic levels of gonadotropin-inhibitory hormone (GnIH) transcript during postnatal development are unknown in primates, we also quantified messenger RNA (mRNA) levels of *RFRP*, a gene encoding GnIH. Moreover, the transcript levels of kisspeptin, a well-known regulator of the hypothalamic neurohormonal axis controlling reproduction, were also checked. Transcript and protein levels of DMXL2 and *Kiss1* transcript levels increase from the newborn to the infantile and from the juvenile (prepubertal) to the pubertal and the postpubertal period. We also noted a clear upsurge in *RFRP* transcript levels in the prepubertal period. In conclusion, the hypothalamic expressions of *Kiss1* and *DMXL2* mRNA increase during infantile, pubertal, and adult stages compared to newborn and juvenile stages in common marmoset monkeys. In contrast, the expression of *RFRP* mRNA upsurges in juvenile monkeys. Further mechanistic studies are needed to characterize the potential inhibitory role of the GnIH-GPR147 signaling in the prepubertal period and the role of DMXL2 in the molecular cascade regulating the neuroendocrine reproductive axis in primates.

**Electronic supplementary material:**

The online version of this article (doi:10.1007/s12035-016-0329-x) contains supplementary material, which is available to authorized users.

## Introduction

Reproductive function is regulated by various neural and endocrine factors of the hypothalamic-pituitary-gonadal (HPG) axis [1]. Among these factors is the gonadotropin-releasing hormone (GnRH), a decapeptide secreted in a pulsatile manner by a specialized set of hypothalamic GnRH neurons. GnRH is a central player controlling downstream pituitary gonadotropins, i.e., the luteinizing hormone (LH) and follicle-stimulating hormone (FSH) synthesis and secretion. Although the GnRH neuronal network is anatomically established in newborn (NB) primates, its activities change at various stages of postnatal development [2]. The activity of GnRH neurons is increased in the infantile stage at minipuberty*—*the time at which an adult-like level of gonadotropins and gonadal sex hormone levels in infants occurs *—*and is lowered again in the juvenile stage due to central inhibition [2, 3]. The GnRH neuronal activities again resurged in true puberty after removal of the central inhibition and then remain at high levels throughout adult reproductive life [2].

The neurobiological mechanism of the onset of puberty is still incompletely known. The most important unanswered questions are as follows: Which signal stimulates the GnRH neurons at the time of minipuberty and true puberty? And what causes the temporary central inhibition of GnRH neurons during the juvenile period demarcating minipuberty and true puberty? Over the years, various upstream regulators of GnRH neurons have been identified and implicated in the control of puberty onset [2]. Among others, glutamate, gamma-aminobutyric acid (GABA), neuropeptide Y (NPY), and neurokinin B were identified as upstream regulators of GnRH neurons [2, 4, 5]. In the last decade, comprehensive studies have also uncovered kisspeptin and GnIH as major regulators of the GnRH neuronal network in vertebrates, including primates [6–9].

In primates, kisspeptin has been implicated in the activation of the GnRH neuronal network at both minipuberty and true puberty in rhesus macaques [7, 8, 10–13]. In human subjects, inactivating mutations in *KISS1R* or *KISS1* caused delayed or absence of puberty, while an activating mutation in *KISS1R* resulted in precocious puberty [14–17]. The kisspeptin system is also involved in the control of adult reproduction [6, 18–21]. In contrast to kisspeptin, GnIH exerts a negative impact on reproductive function [9]. Nonetheless, it is not known whether GnIH has a role in the timing of primate puberty.

The set of players co-orchestrating the neuroendocrine activation of puberty and reproduction is continuously expanding. One of the most recent findings was rabconnectin-3α (aka Dmx-like 2 (DMXL2)), a synaptic vesicular protein, with a possible role in neuroendocrine regulation of reproductive function [22]. Recently, a study reported a homozygous deletion mutation in *DMXL2* in human subjects exhibiting reproductive impairment along with other endocrinopathies and mental retardation [22]. Moreover, haploinsufficiency of *Dmxl2* resulted in a significant loss of GnRH neurons with associated infertility in mice [22]. Besides other regions of the hypothalamus, the localization of rabconnectin-3α was reported in exocytotic vesicles in GnRH neurons. This protein is also expressed in pituitary gonadotropes and endocrine pancreas [22].

In the present study, we aimed to further characterize the role of DMXL2 and GnIH in the regulation of puberty in primates. We used the common marmoset monkey (*Callithrix jacchus*) because this species has many biological and practical advantages compared to other non-human primate (NHP) species [23]. Marmosets also exhibit minipuberty and true puberty [23, 24]. Therefore, marmosets are increasingly used as a NHP model in neurobiology and reproductive research [25, 26]. In the common marmoset, the infantile period lasts from the first week to 3 months, the juvenile period from 3 to 6–8 months, and the pubertal period from 9 to 16–18 months of age [24, 27, 28]. In the present study, we quantified the expression of *DMXL2* and *RFRP* at five stages of postnatal development in common marmoset monkeys: in the NB, the infantile, the juvenile, the pubertal, and the adult stage. As the temporal expressions of the *Kiss1* and *Kiss1r* genes have also not been studied yet, relative to the onset of marmoset puberty, we also included hypothalamic transcript level analyses of these key reproductive function regulator genes in our study.

## Material and Methods

### Marmoset Monkey Hypothalamic Tissue Samples

For this study, hypothalami or whole brains of 18 NB, 5 infantile, 6 juvenile, 6 pubertal, and 18 adult animals were analyzed. These tissue samples were obtained from the primate tissue banks of the Deutsches Primatenzentrum (DPZ), Göttingen, Germany, and the Center of Reproductive Medicine and Andrology (CeRA), University of Münster, Germany. The DPZ is registered and authorized by the local and regional veterinary governmental authorities for primate breeding and research (reference number 122910.3311900, PK Landkreis Göttingen). The hypothalami were dissected by two coronal cuts: one carried out at the level of mammillary bodies and other caudal to the optic chiasm and two parasagittal cuts at the lateral side of hypothalamus. The hypothalamus was cut into two hemi-hypothalami. One of the hemi-hypothalami was used for real-time quantitative polymerase chain reaction (qPCR) analysis and the other for western blotting or immunohistochemistry. The anatomy of the common marmoset brain was determined according to a marmoset brain atlas [29].

### Experimental Design

In this study, expression profiles of *DMXL2*, *Kiss1*, *Kiss1r*, *RFRP*, and *GPR147* mRNAs were analyzed in neonatal (NB, 1–3 days), infantile (2 months), juvenile (5 months), pubertal (15 months), and adult (>5 years) stages via real-time qPCR. There were three to five monkey hemi-hypothalamic samples in the NB, the juvenile, the pubertal, and the adult groups per sex, as well as three samples in the female infantile and two samples in male infantile group. In addition to *DMXL2* mRNA analyses, its protein expression profiles were determined by western blotting. For western blot analysis, three to five samples were used from each of the different postnatal developmental stages in female marmosets and adult male marmosets. Furthermore, immunohistochemical detection of DMXL2 was carried out in NB female and adult male and female marmoset hypothalamic or whole brain sections. The adult female monkeys were in the follicular phase of the ovarian cycle.

Moreover, *DMXL2* and *Kiss1* mRNA expression profiles were also checked in anterior and posterior hypothalami of both male and female monkeys at NB and adult stages (*n* = 4/group). The adult female monkeys were in the follicular phase of the ovarian cycle. For this experiment, the hypothalamus was divided into anterior and posterior parts as has been done by Quennell et al. [30] in the mouse. The anterior part contains the preoptic area, which is located rostral of the optic chiasm, while the posterior part contains the arcuate nucleus (ARC, aka infundibular nucleus in human) along with other nuclei.

### Immunohistochemistry on Sectioned Tissues

Immunohistochemical (IHC) staining of whole hypothalamic/brain sections was carried out as documented elsewhere [31]. After collection, hypothalamic tissues were kept overnight in Bouin’s solution for fixation. After at least 2 days of washes in 70% EtOH, the tissues were embedded in paraffin and 5-μm-thick sections were cut using a microtome. These sections were mounted on slides. In addition to Bouin’s fixed tissues, some paraformaldehyde-fixed brain tissues were also used for IHC. After deparaffinization and rehydration of the tissue sections, antigen retrieval was done by cooking the tissue sections in a microwave oven in 10 mM citrate buffer for 7 min. After cooling down the tissue sections, they were washed for 5 min in wash buffer. After washing, endogenous peroxidase was blocked by incubation with a peroxidase blocking reagent (DakoCytomation Carpinteria, CA, USA, LSAB+ system HRP, K0679). The DMXL2 antibody was purchased from Sigma (cat. no. HPA039375). It was used at a 1:100 dilution for IHC. The dilution of antibody was done in Tris-buffered saline plus 5% bovine serum albumin (BSA). In all incubation steps, the slides were placed horizontally in a humid plastic chamber. The incubation of tissue sections with the primary antibody was carried out overnight at 4 °C, while the other incubation steps were performed at room temperature. Primary antibody on tissue sections was detected using a biotinylated second antibody polymer and horseradish peroxidase (HRP)-conjugated streptavidin from Dako (DakoCytomation Universal LSAB Plus-kit). The chromogenic substrate for HRP was 3,3′-diaminobenzidine. Mayer’s hematoxylin was used for counterstaining of tissue sections. Non-specific rabbit immunoglobulin G (IgG, dilution 1:1000) was used at the same protein concentration as the primary antibody for control staining. The CRI Nuance multispectral imaging camera in conjunction with the Zeiss Axioskop microscope was used to capture images.

### Real-Time Quantitative PCR

For extraction of total RNA from hypothalamic tissues, a NucleoSpin RNA plus kit (Macherey-Nagel GmbH, Düren, Germany) was used according to the manufacturer’s instructions. For the removal of genomic DNA from isolated RNA samples, a DNA-free™ DNA removal kit (AM1906, Ambion, Life Technologies) was used. Using the Omniscript RT kit 200 (Qiagen), 1000 ng of the extracted total RNA was reverse transcribed into complementary DNA (cDNA). The cDNA was diluted with deionized water to a final concentration of 15 ng/μL and stored at −20 °C. A StepOnePlus System (Applied Biosystems, Carlsbad, USA) was used for real-time qPCR analysis. For each PCR reaction, cDNA transcribed from 15 ng mRNA served as a template. The reactions were performed in volumes of 20 μL, containing 1 μL cDNA, 10 μL power SYBR green PCR master mix (Applied Biosystems), 5 μL of forward + reverse primers, and 4 μL nuclease-free water. The sequence of all primers is given in Table 1. qPCR amplification for each sample was carried out in triplicates. Due to less variation in expression of 18S ribosomal RNA in whole hemi-hypothalamic samples during various postnatal developmental stages, the expression value of each gene in each sample was normalized to the corresponding value of 18S rRNA. For normalization of anterior and posterior hypothalamic expression of *Kiss1* and *DMXL2*, beta-actin was used because of stable expression. 2^−ΔΔCT^ method was used for calculation of relative quantification.

**Table 1:** List of primers used for real-time qPCR with ensemble transcript ID or NCBI accession number

Gene	Transcript ID/accession no.	Primer sequences (5′ -3′)	
DMXL2	ENSCJAT00000002098	Forward	CAAGTCAGTTGTGTGGAGTGT
		Reverse	CCACTGGCACTTGAGTTGAC
Kiss1	ENSCJAT00000032872	Forward	ATCAGACATGGCTCCTGTGG
		Reverse	CACAGCGCACGGATTATCC
Kiss1r	ENSCJAT00000042761	Forward	CATCCAGCTGTTCCTAACGC
		Reverse	CAGTTGCTGTAGGACATGCC
GnIH	ENSCJAT00000015396	Forward	CCACATCGAGCTTGTTAACATC
		Reverse	TGTTGACTGCAGGTGTACTCA
GPR147	ENSCJAT00000031793	Forward	TGGTCTGTTTCATCGTGCTC
		Reverse	CTCATCTTGCATGTGGCATT
18S rRNA	AB571241	Forward	CGCGGTTCTATTTTGTTGGT
		Reverse	AGTCGGGCATCGTTTATGGTC
ß-Actin	DD279463	Forward	GATGGTGGGCATGGGTCAGAA
		Reverse	AGCCACACACGCAGCTCGTTGT

### Western Blot Analysis

Protein from ∼20 mg tissue was isolated using the Qproteome nuclear protein kit from Qiagen according to their instruction protocol. This kit is suitable for the isolation of both cytoplasmic and nuclear proteins. The isolated protein fractions were immediately frozen in liquid nitrogen and then stored at −80 °C until used for further analysis.

Concentration of protein in each sample was determined with Bradford assay (Bradford Protein Assay Kit, Pierce) at 592 nm on a spectrophotometer. Twenty micrograms of total protein was diluted in 4× Laemmli buffer (Sigma-Aldrich) and separated by SDS-PAGE in a 12.5% polyacrylamide gel (20 V for 90 min). To check separation and transfer quality, two molecular mass markers of 10–260 kDa, Novex Sharp prestained (cat. no. LC5800) and Magic Markers (cat. no. LC5602, Invitrogen, USA), were applied to the gel. After electrophoresis, the proteins were transferred from gel onto a polyvinylidene fluoride (PVDF, Amersham Hybond P 0.2 PVDF) membrane in a semi-dry blotting system (200 V for 60 min). After this, the PVDF membrane was washed in PBS-T (1× PBS with 0.1% Tween-20) and blocked for 60 min in 5% skimmed milk powder in PBS-T. After washing with PBS-T, the membrane was incubated overnight under agitation at 4 °C with specific DMXL2 (cat. no. HPA039375, Sigma) and β-actin (sc-1616-R, Santa Cruz Biotechnology) primary antibodies. These antibodies were diluted in 5% BSA-added PBS-T. The dilution of DMXL2 antibody was 1:500, while β-actin antibody dilution was 1:1000. Incubation with secondary HRP-conjugated antibody (goat anti-rabbit (R&D no. HAF008), 1:1000 dilution in PBS-T) was done for 1 h at room temperature. Images of protein bands on the membrane were captured using a chemiluminescence reaction kit (enhanced chemiluminescence (ECL), GE Healthcare) and an Intas ChemoCam Imaging System. The optical density of each ECL-detected protein band of both DMXL2 and β-actin was quantified by ImageJ software (NIH). The DMXL2 results are expressed in an arbitrary unit of their ratio to β-actin (internal control). Both DMXL2 bands were quantified separately as well as together and normalized to β-actin. The specificity of DMXL2 bands was tested by preadsorption of the primary antibody with DMXL2 PrEST antigen (cat. no. APREST81430, Sigma-Aldrich Co.; 1:20 ratio). This antigen was used for the generation of the DMXL2 antibody.

### Statistical Analysis

The statistical comparison was done with Student’s *t* test (between two groups) and a one-way ANOVA with post hoc Tukey test (between more than two groups) by using GraphPad prism software (GraphPad Software Inc., La Jolla, CA). All data are presented as means ± SEM. Statistical significance level was set at *P* ≤ 0.05, and different combinations of asterisks and letters indicate statistical significance.

## Results

### *DMXL*2 mRNA and Protein Profiles at Various Stages of Postnatal Development

**Fig. 1:**
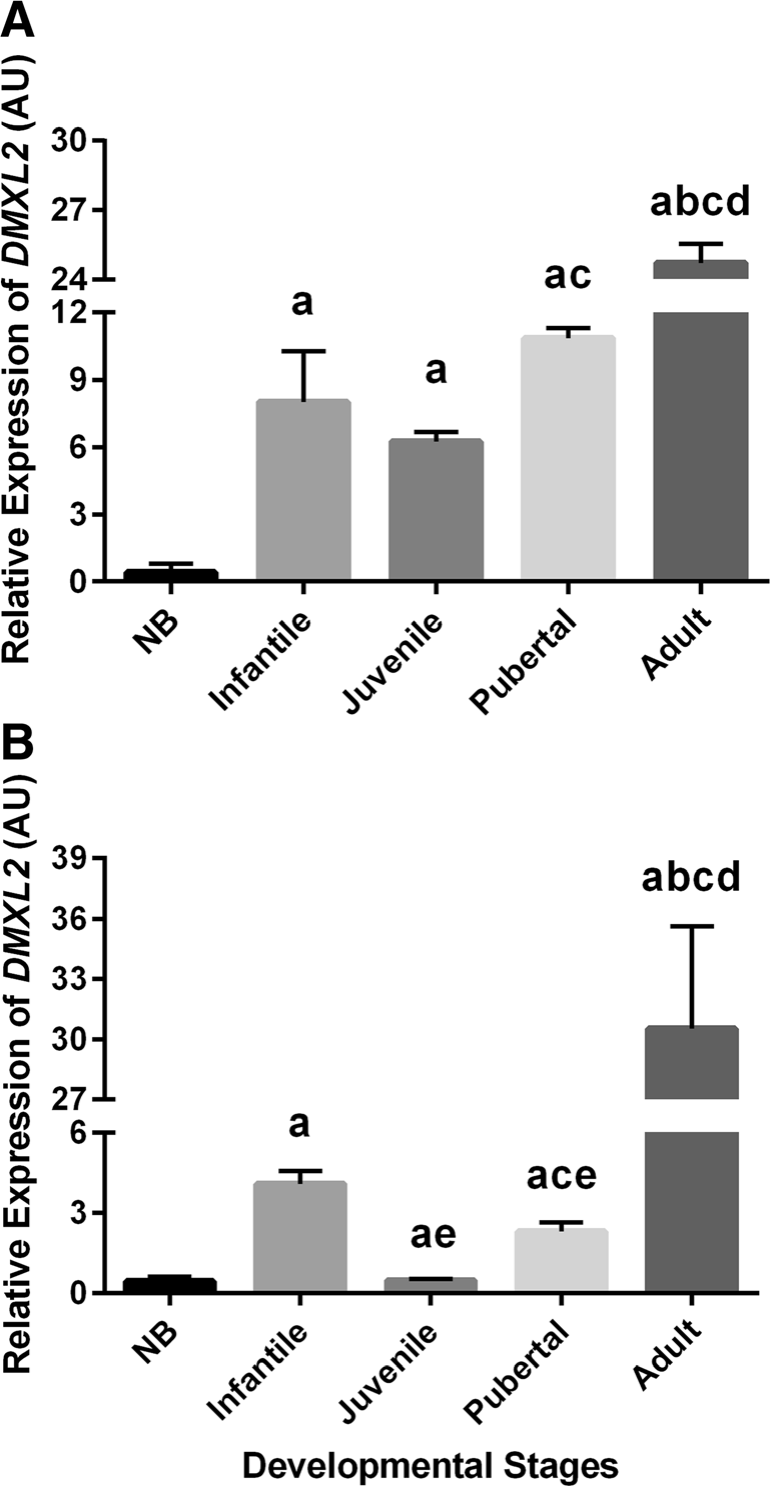
Hypothalamic mRNA expression profiles of *DMXL2* in various developmental stages of female and male marmosets. Hypothalamic *DMXL2* mRNA expression significantly increased from the NB to the infantile and from the juvenile to the pubertal stages in both females (**a**) and males (**b**). Likewise, *DMXL2* transcript levels in hypothalami of both female and male adult marmosets significantly increased as compared to juvenile and pubertal monkeys. Values are expressed as the mean ± SEM. (^a^ *P <* 0.05–0.005 increase vs NB, ^b^ *P <* 0.05–0.01 increase vs infantile, ^c^ *P <* 0.05–0.01 increase vs juvenile, ^d^ *P <* 0.05–0.01 increase vs pubertal, ^e^ *P <* 0.05–0.01 decrease vs infantile; Student’s *t* test and ANOVA followed by post hoc Tukey test)

The relative expression levels of *DMXL2* mRNA in the hypothalami of monkeys of different developmental stages are shown in Fig. 1. In female marmosets, the *DMXL2* transcript levels significantly increase from the NB to the infantile stage (Fig. 1a; *P* < 0.01). A decrease, although not statistically significant in the present sample size, is observed from the infantile to the juvenile stage. More strikingly, a statistically significant increase in *DMXL2* mRNA expression levels was noted from juvenile to pubertal (*P* < 0.05) and adult (*P* < 0.01) monkeys. Likewise, in male marmosets, *DMXL2* mRNA increases from the NB to the infantile stage and from the juvenile to the pubertal and adult stage. Also, the decreasing trend in hypothalamic *DMXL2* mRNA from the infantile to the juvenile stage seen in females was encountered in male marmosets (Fig. 1b). In female monkeys, one-way ANOVA followed by post hoc Tukey test showed that the hypothalamic *DMXL2* transcript levels at all other postnatal developmental stages were significantly (*P* < 0.05) higher as compared to NB. In male monkey, *DMXL2* mRNA levels at pubertal and adult stages were significantly (*P* < 0.05) higher than in NB, while no clear change between NB and juvenile *DMXL2* profiles was encountered. Altogether, hypothalamic *DMXL2* levels increase more than 50-folds from the neonatal period to adulthood with an intermediate peak in infancy in both sexes.

**Fig. 2:**
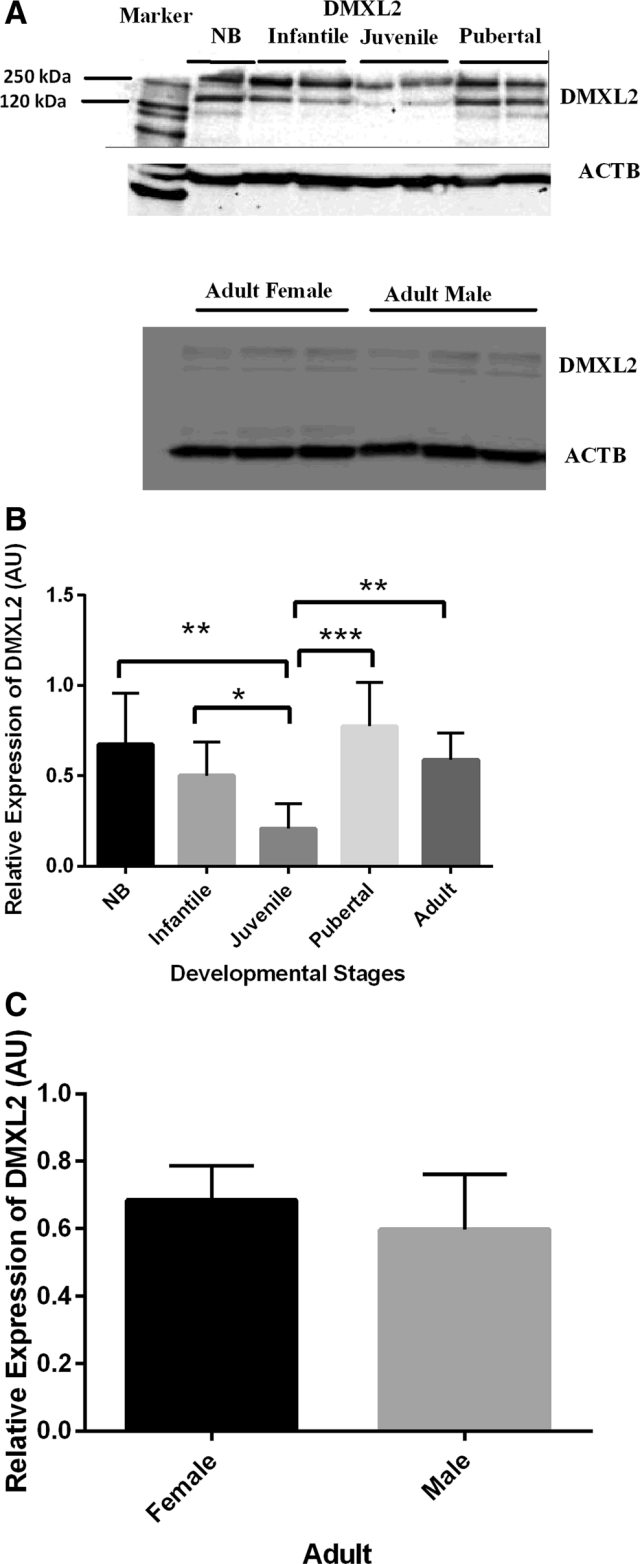
Hypothalamic DMXL2 protein expression profiles in various developmental stages of female and the adult male marmosets. **a** Representative cropped photomicrographs showing DMXL2 western blots in various postnatal developmental stages and in adult female and adult male marmosets. **b** Quantification of DMXL2 protein expression in different postnatal development in female marmosets. Hypothalamic DMXL2 expression in female marmosets significantly decreased from the infantile to juvenile stage and increased from the juvenile to pubertal stages in female monkeys. **c** Comparison of DMXL2 expression in adult female and male marmosets. There was no significant difference in DMXL2 protein levels between adult female and male monkeys. (*P < 0.05, **P < 0.01, ***P < 0.005; Student’s *t* test and one ANOVA followed by post hoc Tukey test)

Hypothalamic DMXL2 protein quantification by western blotting is shown in Fig. 2. Two splice variants giving rise to marmoset DMXL2 isoforms of 340 and 270 kDa have been suggested (http://www.ensembl.org/Callithrix_jacchus). In our study, we observed two immunoreactive bands for DMXL2 (Fig. 2a). One DMXL2 band of 340 kDa and another band between 120 and 250 kDa were observed and measured. Of note, besides the 340 kDa DMXL2 isoform, a 133 kDa splice variant has also been documented for the human in the Ensemble database (http://www.ensembl.org/Homo_sapiens/Transcript/ProteinSummary?db=core;g=ENSG00000104093;r=15:51447711-51622833;t=ENST00000251076). The lower band in our western blots may correspond with this 133 kDa DMXL2 isoform. Both DMXL2 bands were absent from western blots incubated with DMXL2 PrEST antigen-preincubated DMXL2 antibody (supplementary data Fig. 1), indicating specificity of the DMXL2 bands.

DMXL2 protein concentrations mirrored mRNA levels as a significant increase from the juvenile to pubertal stage was observed in marmoset monkeys (Fig. 2b). However, in contrast to mRNA expression, no increase in DMXL2 protein was observed in adults as compared to the pubertal period but adult and pubertal DMXL2 expression was significantly higher as compared to juvenile monkeys. We also quantified the intensity of both bands separately. The expression of both DMXL2 bands was significantly lowered in juvenile monkeys as compared to pubertal and adult monkeys (supplementary Fig. 2a, b). Moreover, the lower band of DMXL2 was also significantly decreased in juvenile monkeys as compared to NB monkeys (supplementary b). There was no obvious difference in the expression levels of DMXL2 between adult female and male monkeys (Fig. 2c and supplementary Fig. 3).

**Fig. 3:**
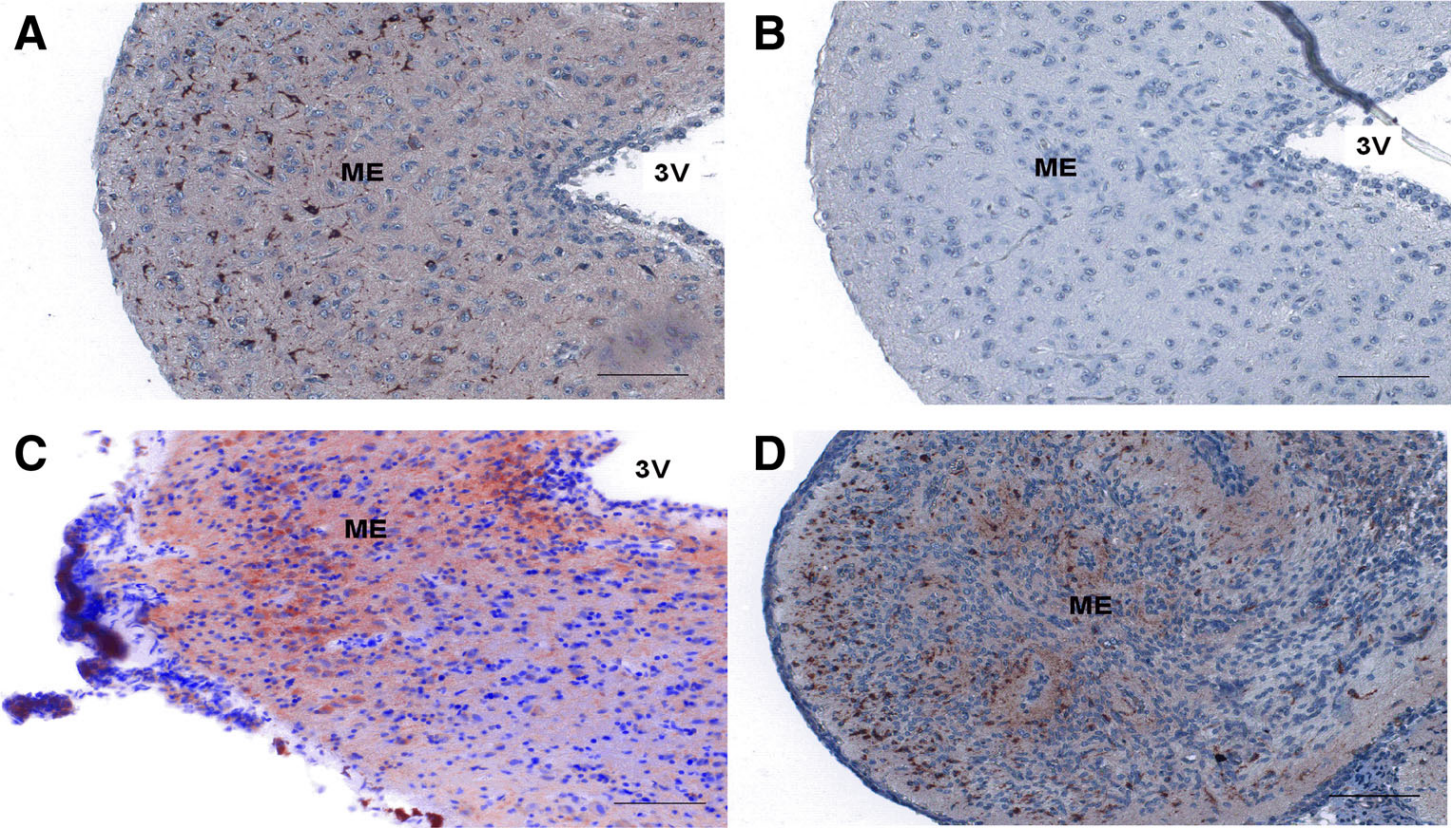
Photographs showing DMXL2 immunostainings in hypothalamus. Immunostaining of DMXL2 was observed in the median eminence region of the adult male common marmoset monkeys (**a**). No staining was observed in primary antibody-omitted (data not shown) or non-specific IgG-treated tissue sections (**b**). Moreover, DMXL2-positive cells were also noted in median eminence of the adult female and NB female common marmoset monkeys (**c**, **d**). *Scale bar* 50 μm, magnification ×40. *ME* median eminence, *3V* third ventricle

A clear immunohistochemical staining of DMXL2 expression was observed in the median eminence of both adult male and female common marmoset monkeys (Fig. 3a and c). Moreover, DMXL2 staining was also observed in median eminence of female NB monkeys (Fig. 3d). No staining was observed in primary antibody-omitted (data not shown) or non-specific IgG-treated tissue sections (Fig. 3b).

### Hypothalamic *Kiss1* and *Kiss1r* mRNA Profiles at Various Stages of Postnatal Development

**Fig. 4:**
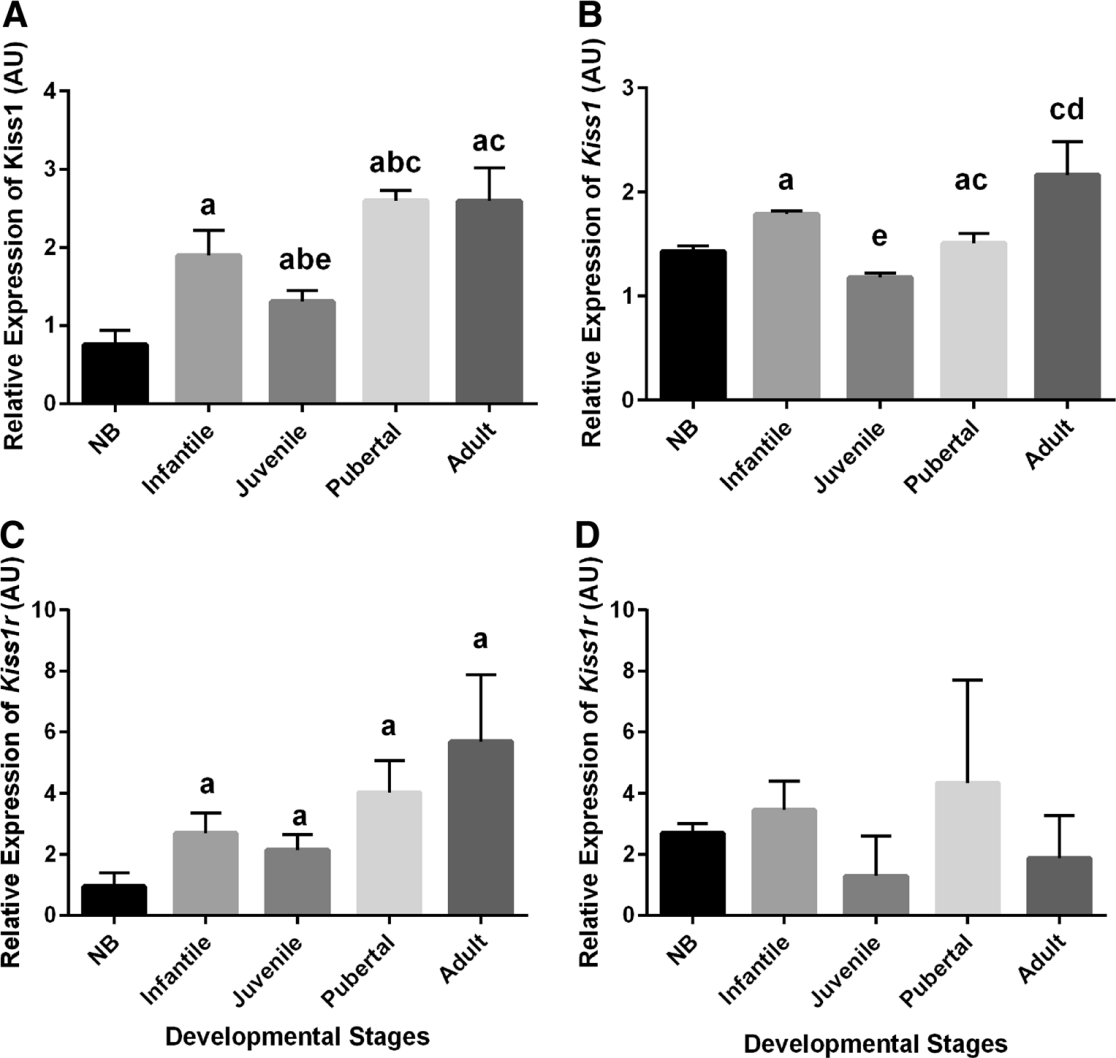
Hypothalamic expression profiles of *Kiss1* and *Kiss1r* mRNA in various postnatal developmental stages in female and male marmosets. **a** Hypothalamic *Kiss1* mRNA expression in female marmosets significantly increased from the NB to the infantile and from the juvenile to the pubertal stage. **b** In male marmosets, hypothalamic *Kiss1* expression also increased in infantile and pubertal/adult marmosets as compared to NB and juvenile marmosets, respectively. **c** *Kiss1r* expression significantly increased from the NB to the infant monkeys, while there was no statistically significant difference in other stages in female marmosets. **d** There was no significant change in the expression of *Kiss1r* between any stages in male marmosets. (^a^ *P <* 0.05–0.005 increase vs NB, ^b^ *P <* 0.05–0.01 increase vs infantile, ^c^ *P <* 0.05–0.01 increase vs juvenile, ^d^ *P <* 0.05–0.01 increase vs pubertal, ^e^ *P <* 0.05–0.01 decrease vs infantile; Student’s *t* test and ANOVA followed by post hoc Tukey test)

The relative levels of *Kiss1* and *Kiss1r* mRNA in the hypothalamus are shown in Fig. 4. The transcript levels of *Kiss1* were significantly higher in infantile female as well as in male monkeys in comparison to NB (Fig. 4a, b). One-way ANOVA followed by post hoc Tukey test showed a significant (*P <* 0.05) increase in *Kiss1* mRNA profile from NB to all other postnatal developmental stages in the female. Moreover, there was also a statistically significant (*P <* 0.05) increase in *Kiss1* mRNA levels from the juvenile stage to the pubertal and adult stages, respectively, in both sexes. The developmental hypothalamic *Kiss1r* mRNA expression levels basically paralleled the expression levels of the *Kiss1* mRNA (Fig. 4c, d). However, a statistically significant increase in *Kiss1r* expression was detected only from the neonatal to infantile stage in female marmosets, while in the male, no clear differences were encountered when consecutive stages were compared. Interestingly, in contrast to the females, in males, the *Kiss1r* levels declined in adults compared to the pubertal stage. The comparison of the relative *Kiss1r* expression in adult females and males showed no significant differences at the current sample size.

### Hypothalamic mRNA Profiles of *RFRP* (*GnIH*) and *GPR147* (*GnIH-R*) at Various Stages of Postnatal Development

**Fig. 5:**
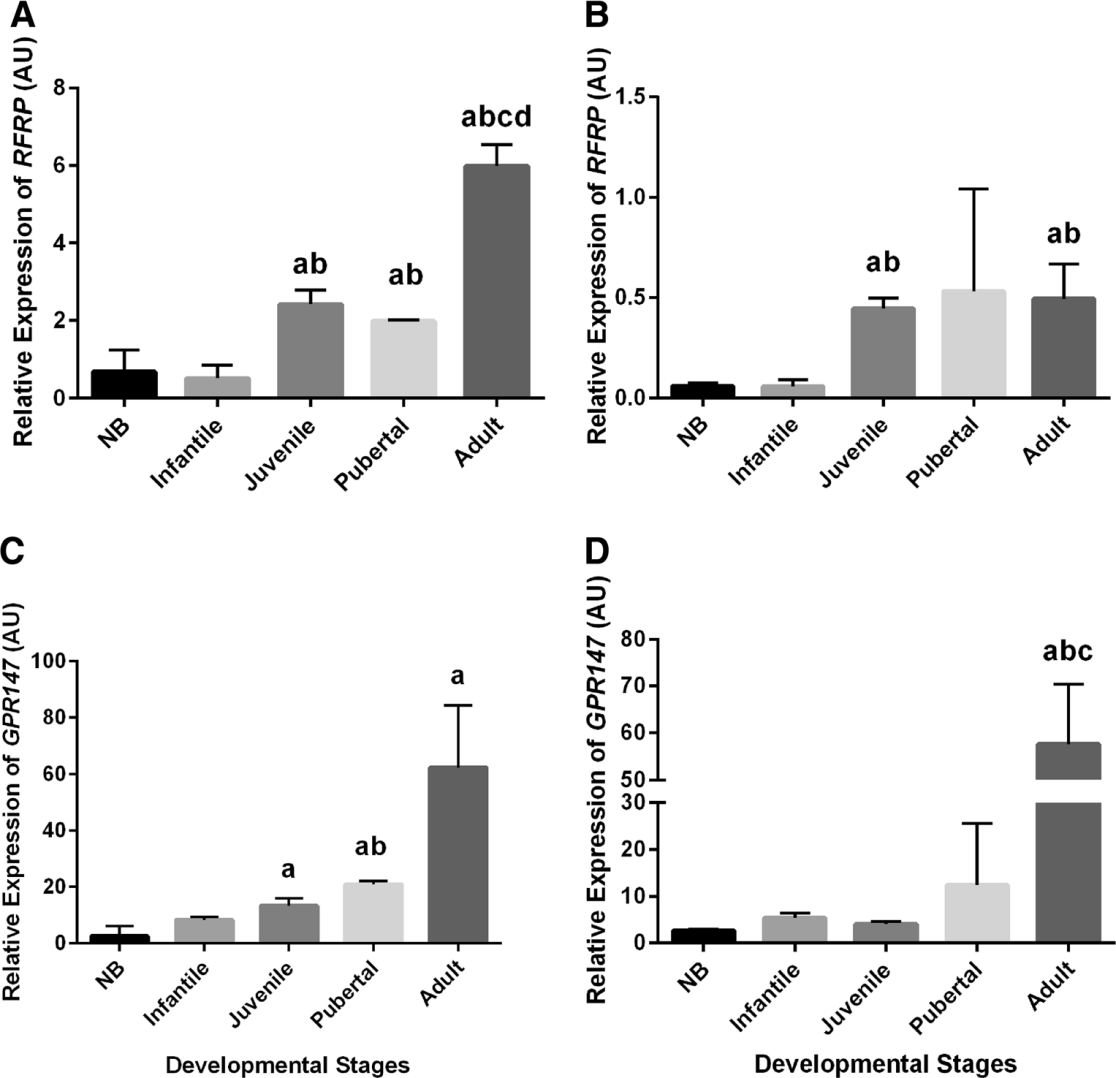
Expression profiles of *RFRF* and *GPR147* mRNA in various postnatal developmental stages in marmoset monkeys. **a** Hypothalamic *RFRP* gene expression in female marmosets significantly increased from the infantile to the juvenile stage. Moreover, *RFRP* transcript levels also greatly increased from the pubertal to the adult stage. **b** In male marmosets, a statistically significant change was observed only from the infantile to the juvenile stage. **c** *GPR147* mRNA expression levels significantly increased in juvenile, pubertal, and adult monkeys as compared to NB female marmosets. **d** In male marmosets, a statistically significant increase was observed only in adult monkeys as compared to NB, infant, and juvenile monkeys. (^a^ *P <* 0.05–0.005 increase vs NB, ^b^ *P <* 0.05–0.01 increase vs infantile, ^c^ *P <* 0.05–0.01 increase vs juvenile, ^d^ *P <* 0.05–0.01 increase vs pubertal; Student’s *t* test and ANOVA followed by post hoc Tukey test)

The relative expression levels of *RFRP* and *GPR147* mRNA in the marmoset hypothalami are shown in Fig. 5. In both males and females, the transcript levels of the *RFRP* gene were significantly raised from the infantile to the juvenile stage. In male marmosets, there was no significant change in the expression of *RFRP* mRNA between juvenile and pubertal as well as adult stages. In female marmosets, there was no significant alteration in the abundance of *RFRP* in pubertal monkey hypothalami as compared to juvenile ones. However, a significant increase (*P* < 0.05) was observed from the pubertal to the adult stage. There was no clear change in expression of *GPR147* in any of the postnatal developmental stages when directly compared to the preceding stage. However, when neonatal and adult expression levels were compared, we found a significant increase in adult levels for both males and females (*P* < 0.05). In the females, juvenile and pubertal *GPR147* expression levels were also significantly (*P* < 0.05) higher than in the neonatal stage. Moreover, in male adults, the *GPR147* transcript level was also significantly higher than infantile and juvenile transcript levels.

### Expression Profiles of *DMXL2* and *Kiss1* in Anterior and Posterior Hypothalamus of NB and Adult Monkeys

**Fig. 6:**
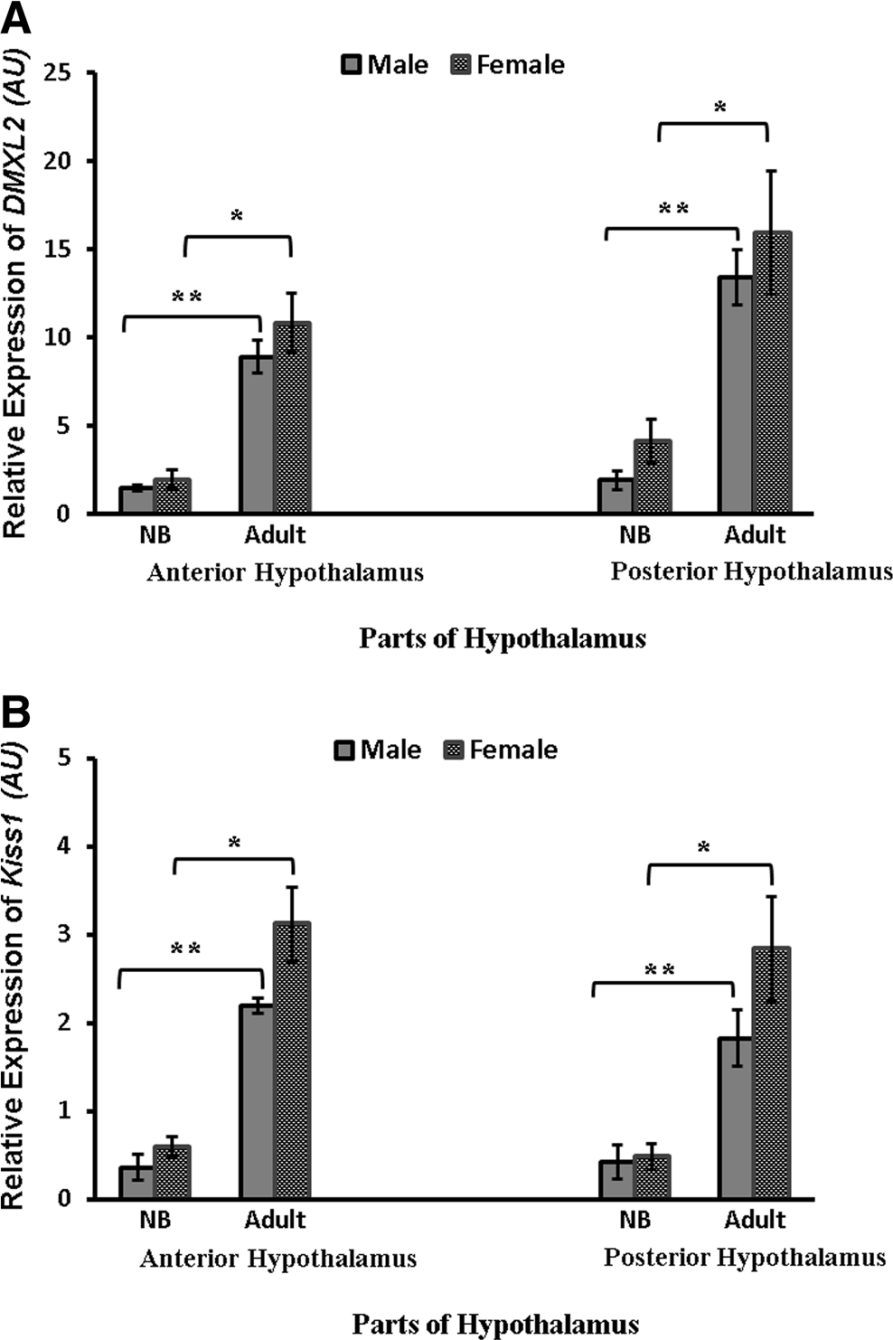
Expression profiles of *DMXL2* and *Kiss1* mRNA in anterior and posterior hypothalamus in NB and adult marmoset monkeys. **a** *DMXL2* gene expression in both male and female adult common marmosets significantly increased in the anterior as well as in the posterior hypothalamus as compared to NB. **b** *Kiss1* mRNA expression was also significantly increased in the anterior and posterior hypothalamus of both male and female adult monkeys as compared to NB. (*P < 0.05, **P < 0.01; Student’s *t* test)

The transcript abundances of *DMXl2* and *Kiss1* were significantly increased (*P* < 0.05–0.01) in both the anterior and posterior hypothalamus of adult common marmosets as compared to NB monkeys in both sexes (Fig. 6). No significant difference was observed at the current samples size between the transcript levels of female and male monkeys as well as between anterior and posterior hypothalamic levels at both the NB and the adult stage.

## Discussion

**Fig. 7:**
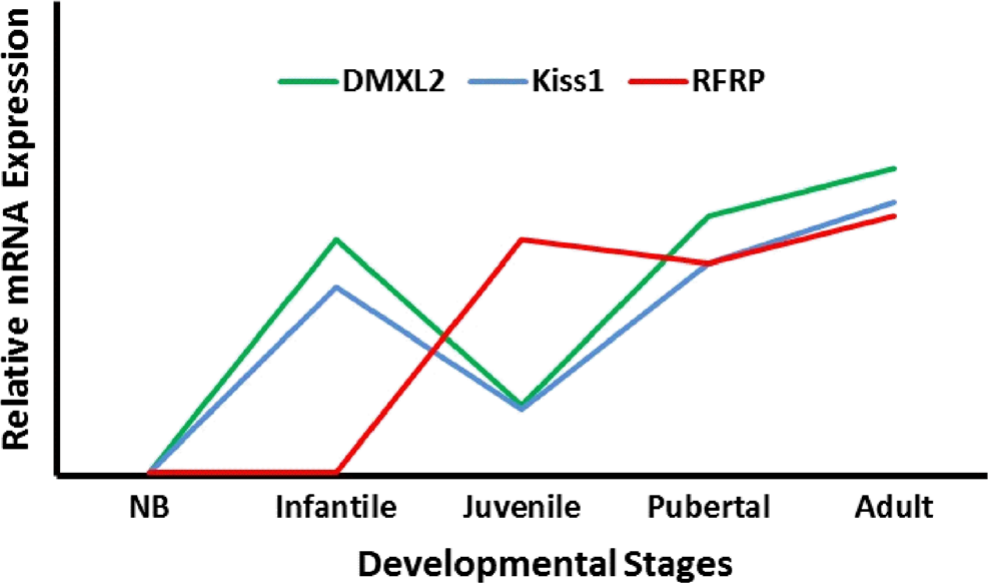
A graphical summary of *DMXL2, Kiss1*, and *RFRP* mRNA expression patterns in different postnatal developmental stages in marmoset monkeys

The hypothalamic neural circuitry controlling reproductive function is very complex [1, 2]. A large number of molecular factors constitute this network. Some of these factors inhibit GnRH neurons, while others stimulate them [1, 2]. A very recent addition to the network of hypothalamic factors involved in the GnRH release is DMXL2 [22]. In this study, we took advantage of the availability of very rare primate hypothalamic samples of different postnatal developmental stages. To initially characterize alterations in DMXL2 expression at various postnatal developmental stages in primate, the quantification of mRNA and protein levels of DMXL2 was carried out in hypothalami of common marmoset monkeys at neonatal, infantile, juvenile, pubertal, and adult stages. A robust increase in the expression of DMXL2 transcript was noted from the neonatal to the infantile stage as well as from the juvenile to the pubertal stage (summarized in Fig. 7). This increase mirrors heightened *Kiss1* expression during minipuberty and actual puberty in infant and adult marmosets, respectively. Additionally, an increased expression of the *RFRP* gene, encoding GnIH, was observed in the juvenile period compared to the infantile period.

It is well known that the reproductive axis is at least transiently active in infant primates [2]. The activity of the GnRH secretory system increases tremendously at minipuberty in the infantile period [3, 8]. In marmosets, the infantile period lasts from the first week until 12–15 weeks of age [24, 27, 28]. During the juvenile period, the activity in the GnRH secretory system is again reduced. Hence, the reproductive axis enters a dormant phase. A central inhibition has been suggested to be responsible for suppression of GnRH release during the prepubertal juvenile period [2]. The GnRH secretory system again activates during true puberty.

Studies in other primate models suggest a role of kisspeptin in the augmentation of the hypothalamic GnRH neuronal network during minipuberty in the infantile period and true puberty [10–13]. Therefore, our results of increased *Kiss1* expression in infant and postpubertal marmosets are consistent with those of previous studies and suggest that kisspeptin also plays an important role in the regulation of the neuroendocrine reproductive axis, at both minipuberty and true puberty, of common marmoset monkeys.

Studies in various mammalian species reported two distinct populations of kisspeptin expressing neurons in the hypothalamus: one in the preoptic area (POA) of the anterior hypothalamus and another in the ARC of the posterior hypothalamus [32, 33]. In rodents, the POA kisspeptin population is present in the anteroventral periventricular nucleus (AVPV) and adjacent periventricular area [32, 33]. A significantly higher *Kiss1* mRNA expression has been reported in AVPV in comparison to ARC in rodents. Moreover, a sex-specific difference in *Kiss1* expression has also been documented in these animals [32–34]. We have not observed a significant difference in *Kiss1* mRNA expression between the anterior and posterior hypothalamus as well as between male and female monkeys. This discrepancy may be due to species differences because the anterior hypothalamic *Kiss1*-expressing cell population is more scattered and less numerous in rhesus monkeys and sheep than in rodents [35]. As these two populations of kisspeptin neurons are differentially regulated by sex steroids, the analysis of *Kiss1* mRNA expression of these two populations during minipuberty and true puberty in primates would be worth studying. However, in the present study, this could not be analyzed due to a shortage of samples of the corresponding stages.

The most important finding of the present study is a clear increase in DMXL2 expression during minipuberty and true puberty. DMXL2 is likely a participant in a cascade of molecular events that boosts the performance of the neuroendocrine reproductive axis at both minipuberty and actual puberty in the marmoset monkey. However, the hierarchical position of DMXL2 in this biochemical cascade regulating the reproductive axis is not clear. Besides other brain areas, DMXL2 is expressed in various regions of the hypothalamus, including the GnRH neurons [22]. In the GnRH neurons, DMXL2 is present in exocytotic vesicles [22]. These findings suggest that it may play a role in the secretion of GnRH. However, currently, it is not known whether DMXL2 is a marker of puberty or only GnRH release. Available evidence favors the latter notion [22]. It is a well-documented fact that during the juvenile period, GnRH secretion is significantly decreased [2]. Therefore, it is possible that decreased GnRH secretion might lead to reduction in DMXL2 abundance.

The importance of DMXL2 for the reproductive system in general and for GnRH neurons specifically is suggested by a recent study by Tata et al. [22]. They conditionally deleted *Dmxl2* from neurons of mice. Haploinsufficiency of *Dmxl2* results in a significant loss of GnRH neurons causing delayed puberty and a clear reduction of reproductive output. Moreover, they also reported a homozygous deletion mutation in *DMXL2* in human subjects of reproductive impairment [22]. Nevertheless, further studies are required to reveal whether blocking DMXL2 expression affects GnRH secretion or not. It is noteworthy that blocking DMXL2 also causes impairment of the endocrine functions of the pancreas [22]. An in vitro analysis showed that knockdown of *Dmxl2* in an insulin-secreting cell line led to impairment of insulin secretion [22].

Another important observation of this study is a clear upsurge in expression of *RFRP* during the prepubertal period. This finding suggests that GnIH might be involved in central inhibition by restraining pulsatile secretion of GnRH during the prepubertal period. This hypothesis is supported by evidence that GPR147 is expressed on GnRH neurons [36, 37]. Furthermore, administration of GnIH or its mammalian ortholog, RFRP3, has been reported to result in inhibition of GnRH secretion [9, 38–40]. Moreover, GnIH also reduced the firing rate of GnRH neurons in an in vitro analysis [41, 42].

A large body of compelling evidence shows numerous other upstream molecular factors in the postnatal developmental regulation of the neuroendocrine reproductive axis in primates [2]. Besides many others, the most important molecular factors in this regard are kisspeptin, GABA, glutamate, NPY, and astroglial factors [2, 7, 8]. Therefore, in the future, it will be important to decipher how DMXL2 and GnIH are linked with the known regulators of the GnRH neuronal network.

In summary, our results demonstrate a significant increase in the abundance of *DMXL2* and *Kiss1* during infantile and postpubertal adult stages compared to the respective preceding developmental stages in a non-human primate species. In contrast, the *RFRP* transcript levels were heightened in juvenile period. However, more comprehensive studies are needed to further characterize functionally the role of kisspeptin-Kiss1r signaling in the activation of the reproductive axis during minipuberty and true puberty in the marmoset and a potential inhibitory role of the GnIH-GPR147 pathway in the prepubertal period. Moreover, mechanistic experiments on the role and hierarchical position of DMXL2 in the molecular cascade regulating the neuroendocrine reproductive axis in primates are also required.

## Electronic supplementary material

ESM 1 (DOCX 231 kb)
