# Supplementary material for: Dynamic Regulation of Hypothalamic DMXL2, KISS1, and RFRP Expression During Postnatal Development in Non-Human Primates
Source: Mol Neurobiol. 2016 Dec 12;54(10):8447–57. doi: 10.1007/s12035-016-0329-x (PMC5684250; doi:10.1007/s12035-016-0329-x)
Supplement: Supplementary file 1 — (DOCX 231 kb) [file 12035_2016_329_MOESM1_ESM.docx]

**Dynamic regulation of hypothalamic *DMXL2, KISS1*, and *RFRP* expression during postnatal development in non-human primates**

**Fazal Wahab**^1*^**, Charis Drummer**^1^**,**  [**Stefan Schlatt**^2^](http://humrep.oxfordjournals.org/search?author1=Stefan+Schlatt&sortspec=date&submit=Submit), **Rüdiger Behr**^1*^**.**

^1^Platform Degenerative Diseases, German Primate Center, Kellnerweg 4, 37077 Göttingen, Germany.

^2^Institute of Reproduction and Regenerative Biology, Centre of Reproductive Medicine and Andrology, Albert-Schweitzer-Campus 1, Building D11, 48149 Münster, Germany.

^*^**Correspondence:** [fwahab@dpz.eu](mailto:fwahab@dpz.eu) (FW); rbehr@dpz.eu (RB)


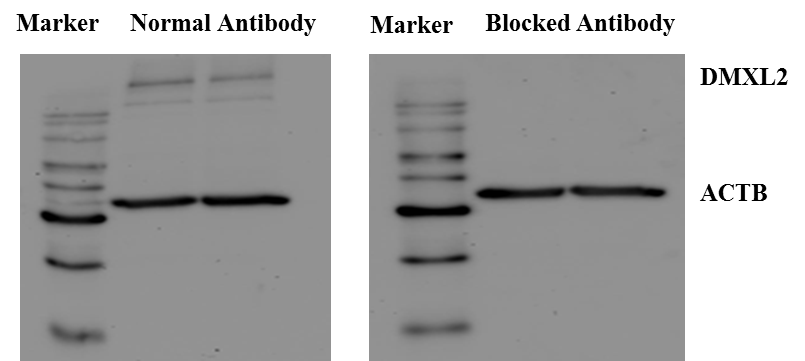


**Figure 1.** DMXL2 bands of two hypothalamic samples of adult female after incubation with normal DMXL2 primary antibody (left panel) and antigen preadsorption blocked DMXL2 primary antibody (right panel). No DMXL2 bands were observed in antigen blocked antibody western blot.

**Figure 2.** Comparison of upper (A) and lower (B) DMXL2 bands in relation to beta-actin during various postnatal developmental stages of female marmoset monkeys.

**Figure 3.** Comparison of upper (A) and lower (B) DMXL2 bands in relation to beta-actin in the adult female and male common marmoset monkeys.
